# Toward the Rational Design of Organic Solar Photovoltaics: Application of Molecular Structure Methods to Donor Polymers

**DOI:** 10.1021/acs.jpca.1c07091

**Published:** 2021-12-14

**Authors:** Sandile Mamba, David S. Perry, Mesfin Tsige, Giuseppe Pellicane

**Affiliations:** † School of Chemistry and Physics, University of Kwazulu-Natal and National Institute of Theoretical and Computational Sciences (NITheCS), 3209 Pietermaritzburg, South Africa; ‡ Department of Chemistry, The University of Akron, Akron, Ohio 44325-3601, United States; § School of Polymer Science and Polymer Engineering, The University of Akron, Akron, Ohio 44325-3909, United States; ∥ Dipartimento di Scienze Biomediche, Odontoiatriche e delle Immagini Morfologiche e Funzionali, Università degli Studi di Messina, 98125 Messina, Italy; ⊥ CNR IPCF, 37-98158 Messina, Italy

## Abstract

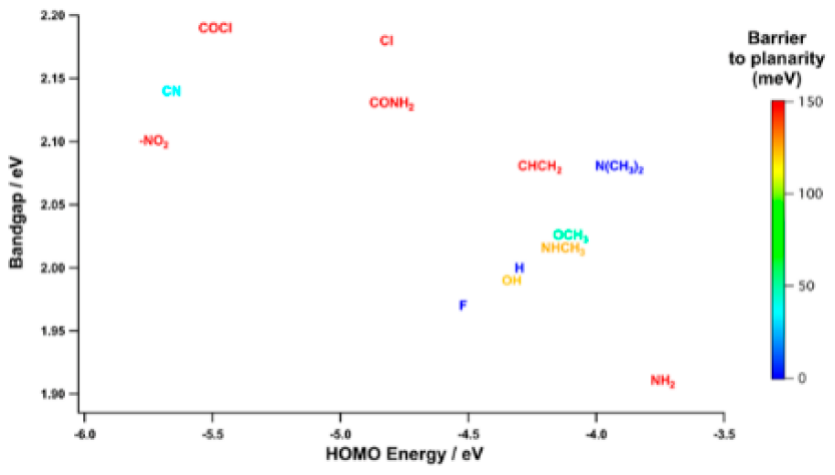

Conjugated polymers are promising candidates in the design of polymer solar cell materials with suitable electronic properties. Recent studies show that the use of different functional groups as side chain in thiophene-based polymers changes the electronic and conformation structures. Here we design new thiophene-based molecules by replacing the hydrogen attached to the backbone of P3MT with electron-donating and electron-withdrawing groups. We then calculate the HOMO, LUMO, and HOMO–LUMO energy gap to quantify the theoretical merit of the new polymers as solar absorbers and their inter-ring torsional potential to understand their suitability to link together in high conductivity, extended conjugated systems. Calculations are done with first-principles density functional theory (DFT), implemented using B3LYP with dispersion function and 6-31G(d,p) as basis set. Our results show that the HOMO–LUMO gap is sensibly lowered by donating groups and we found that the substitution of the hydrogen with −NH_2_, and −F gives an energy gap lower than the energy gap of P3MT. The lowest energy gap was found when substituting with −NH_2_. Electron-withdrawing groups lower the HOMO, with the overall lowest found when −NO_2_ is used. −COCl, −CONH_2_, and −Cl give a steric hindrance greater than that of PTB7, which is set as reference. Our calculations show a possible approach to the rational design of donor materials when substituents are inserted systematically in a generic oligomer.

## I Introduction

Modern technology to harvest solar energy is mainly based on crystalline or polycrystalline silicon, in spite of the high cost of manufacturing semiconductors with that material.^1,2^ An alternative technology based on solution processable thin films emerged as an interesting choice because of the weak absorption of visible light in crystalline Si, which makes solar devices based on this element thicker, rigid, and rather heavy. By contrast, thin films are very light and can be mounted on a flexible substrate, which makes them more adaptable to diverse situations and potentially much cheaper. In fact, they constitute the common denominator for solar devices based on dye-sensitized,^3^ quantum-dot,^4^ organic,^5^ and perovskite^6^ photovoltaics. Perovskite solar cells are the ones with an efficiency similar to crystalline silicon solar cells, but they still show low stability for outdoor applications and potentially exhibit toxicity issues due to lead content.^7^ On the contrary, organic photovoltaics (OP) uses conjugated polymers^8^ as a solar absorber, and besides not needing any inclusion of heavy metals (which is also meaningful from a more general perspective of life-cycle considerations), it also offers several advantages in terms of ease of device processability and cheap material resources.^9−13^ Notwithstanding their advantages, organic solar cells (OSCs) still exhibit a considerably lower power conversion efficiency compared to inorganic ones.^14^ On the other hand, their performance is rapidly improving as a result of intense research activity in the field, e.g., tandem solar cells based on conjugated polymers have recently shown a power conversion efficiency (PCE) of 17.3%.^15^

Organic photovoltaic devices are often composed of a mixture of donor polymers and acceptor organic molecules and their PCE is heavily influenced by the effectiveness of the solar absorber, which is primarily determined by the energy band structure of the molecules. For instance, the difference (*E*_*g*_^*hl*^) between the highest occupied molecular orbital (HOMO) and lowest unoccupied molecular orbital (LUMO) of the organic molecule can be used as an estimate of the energy band gap (*E*_*g*_),^16^ which is the energy difference between the ionization potential and electron affinity of the material. The value of *E*_*g*_ is a lower bound for the range of frequencies of the solar spectrum that can be absorbed by the photoactive material and can potentially be converted into electricity. Conjugated polymers have been intensely explored as donor materials, because of their unique properties, which originate from their extended π-conjugated system delocalized over a large number of recurrent monomer units.^17^ When the electrons of a π-conjugated polymer are excited from the HOMO to the LUMO energy level (EL), they form an exciton (bound electron–hole pair). Then, the exciton can diffuse to the interface with the acceptor organic molecule, where it splits into free charge carriers, which can be transported to the electrodes. The photoactive polymer and the electron acceptor are blended in a bulk-heterojunction (BHJ) configuration, so to maximize interfacial contact and minimize exciton travel distance.^18^ These two conditions in the BHJ configuration are best met when the donor and the acceptor moieties are intermixed on the nanometer scale, which means that the film morphology considerably affects the device performance. While highly ordered donor and acceptor domains could ensure excellent charge transport, this morphology is very difficult to prepare because its formation relies on phase separation of the donor and acceptor materials during the formation of the absorber film.

When the exciton splits at the interface, the electron lands on the LUMO EL of the acceptor molecule, while the hole stays on the HOMO EL of the donor molecule. Fullerene derivates are one of the most common acceptor molecules since they have excellent electron accepting/transporting behavior.^18^ The PCE increases in the presence of a high open circuit voltage (*V*_OC_), which is correlated with the energy gap between the HOMO of the donor polymer and the LUMO of the acceptor, because of the energy loss to overcome the binding energy of an electron–hole pair in the excitons.^19^ Since the LUMO of the widely used fullerene acceptor PC_61_BM is −4.2 eV,^18^ there is an upper bound for the LUMO of the donor polymer, and the PCE can only be maximized by a low HOMO energy of the donor. However, PCE is also maximized by a high short circuit current *J*_*SC*_, that is achieved when a high number of excitons are generated, i.e., when low band gap polymers are used since they are able to absorb visible light in a wide range of frequencies.^18^ Thus, two competing factors often determine the PCE of the active material when fullerene derivates are used. Finally, PCE is also influenced by the fill factor, FF, which depends on the morphology of the photoactive material and reflects the shape of the current–voltage curve.^20^

In the rational design of high-performance conjugated polymers, an efficient way to modify the HOMO and LUMO levels of a π-conjugated polymer system involves the copolymerization of electron-releasing (donor) or electron-withdrawing (acceptor) units that will respectively increase the HOMO level or lower the LUMO one and overall reduce the band gap via internal charge transfer.^17,20^ Most of donor units in semiconducting polymers with high PCE are thiophene derivatives, which also show effective π–π stacking (i.e., long conjugation length) and high hole mobility.^11^ Some efforts toward further optimization of the chemical structure in this family of copolymers were somewhat successful, for example, with narrow-bandgap copolymers as PTB7-Th^21^ and PBDTT-SF-TT,^22^ in which the alkyl group on the thiophene substituents of PTB7-Th was replaced with an alkylthio group, leading to deeper energy levels. Systematic variation of side chains is a way of controlling the properties of conjugated polymers.^23^ On the other hand, attaching different side chains to the polymer backbone may easily cause steric hindrance and twisting of the conjugated backbone, which in turn could lead to a large band gap and low carrier mobility.^20^ The choice of strong or weak electron-donating/accepting substituents on the backbone chain allows us to fine-tune the energy levels and band gap and thereby impact the photovoltaic properties of solar cells. That is especially true in the past few years because a variety of nonfullerene/small molecule acceptors have been designed and proved to be more efficient than fullerene derivatives. For example, the substitution of sulfur with selenode in benzodithiophene (BDT) and thieno[3,4-*b*]thiophene (TT) units of polythiophene derivatives, which belong to the family of poly(3-alkylthiophene)s (P3ATs), lowers its band gap.^24^ Other polythiophene derivatives as donor polymers, including the widely studied regioregular poly(3-hexylthiophene) (P3HT), have been designed and synthesized by attaching electron-withdrawing carboxylate substituents to the side chain,^25^ or fluorine atoms to the backbone.^26^ In a recent study,^27^ the use of a chlorinated acceptor brought the PCE above 16%, which was also supported by density functional theory calculations where the long alkyl side chains were simplified to methyl or ethyl groups to construct the molecular models. Bhatta et al.^28,29^ have investigated the inter-ring torsional potential conformations in P3HT and related polymers and its impact on the properties of donor materials for OP applications.^30,31^ While pure P3HT shows polymer backbones with trans coplanar thiophene rings, i.e., extended conjugation, in the heterogeneous blends of the BHJ (P3HT/PCBM film), the conjugated backbones of P3HT get twisted and different π-conjugated polymer segments will produce different conformations, i.e., torsional disorder. The average effect of the different band gaps of these polymer segments emerges macroscopically as a blue shift of the HOMO–LUMO absorption spectrum^31^ relative to the spectrum of crystalline solid P3HT, resulting in poorer overlap with the solar spectrum, and consequently lower PCE. A significant barrier to coplanarity, or torsional disorder, may also determine hole trapping within π-conjugated donor polymer segments, and higher HOMO orbitals, which can lower the charge conductivity of the active layer of organic solar cells.^30,31^ Of course, torsional or conformational disorder is naturally dependent on the film morphology and also correlated with the PCE and conductivity of the material.

In summary, optical properties and charge transport in the active layer, and ultimately PCE depend on *E*_*g*_, *V*_*OC*_, *J*_*SC*_, and FF, which are then the prime targets for rational design calculations of organic solar photovoltaics, by first-principles methods.^20^ For any given OP system, these quantities depend not only on the identity of the donor and acceptor materials but also on the way the two materials are combined to form the bulk heterojunction (BHJ), which makes their characterization a huge multiscale computational problem. In this paper, we focus on a more limited objective but still relevant to the goal of rational design, that is, to screen candidate donor polymers by insertion of electron-withdrawing and electron-donating substituents. Our aim is to identify properties of single polymer oligomers that will enable the screening of potential high-performance polymer materials and to calculate those properties with density functional theory (DFT). Even though DFT is a well-known and standard computational method for studying the electronic properties of materials, and molecular engineering of conjugated polymers with electron-withdrawing and electron-donating substituents to alter their electronic properties is a molecular design concept that has already been proposed in the literature,^17,20^ a systematic and tractable computational study, applying DFT to donor polymers substituted with electron-donating and electron-withdrawing groups, seems lacking in the literature. Since substituents can considerably change photovoltaic properties of conjugated polymers, and their PCE, we were prompted to try to accomplish this effort in this research work. Also new in this framework is our procedure for screening potential donor polymers for organic photovoltaics. This procedure is based on the elimination, from the list of potential candidates for OP applications, of donor polymers that are not expected to adopt an all-trans coplanar ring structure, i.e., optimal π-conjugation, because noncoplanar structure will likely result in low electrical conductivity in organic solar cells.

Accordingly, we have selected the following three oligomer properties, which should allow many potential donor materials to be eliminated from consideration before they are synthesized in the lab and others to be sorted into more- and less-promising candidates:

(a) The HOMO–LUMO energy gap, *E*_*g*_^*hl*^, which we take as a measure of the bandgap *E*_*g*_ in the bulk polymer material.
(b) The HOMO energy, which, together with *E*_*g*_^*hl*^ and the corresponding properties of the acceptor material, determines the alignment of the donor and acceptor energy levels. As we made clear before, the alignment of the donor and acceptor energy levels in turn impact *V*_*OC*_ and *J*_*SC.*_ Specifically, the LUMO level of the acceptor needs to be lower than the LUMO of the donor to create a driving force for the electron transfer between the two materials, but it should not be too low because that will reduce *V*_*OC*_, and thus impact the PCE.
(c) The barrier to coplanarity between adjacent rings in the oligomer, which impacts the propensity of the oligomers to stack in a planar lamellar structure in the bulk polymer and in the BHJ of a practical device. Such a planar lamellar structure is desirable to promote extended conjugation along the chain of many monomer units and hence high electrical conductivity of the resulting polymer material. The minimization of the steric hindrance to the donor polymer conformation with coplanar rings would allow optimal π-conjugation. Clearly, donor polymers unsuited to extended π-conjugation will not perform well in practical OP devices.

**Figure 1:**
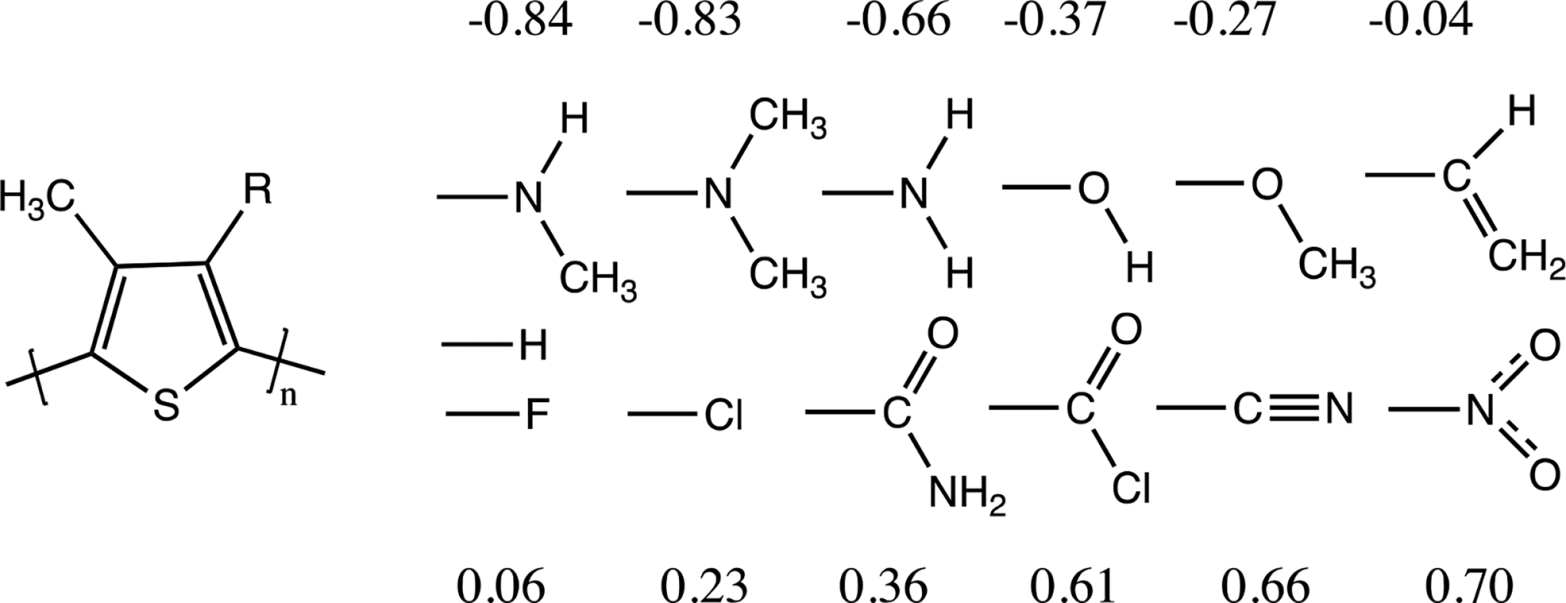
A monomer of P3MT (with R = H) and the tested substituents for R are shown from electron-donating (top row) to electron-withdrawing (bottom row) groups. The numbers are the respective Hammett sigma parameters relative to *H* = 0.^34^

We design oligomers based on poly(3-methylthiophene) by substituting a hydrogen atom of P3MT (see substituent R, shown in Figure 1). Instead of thiophene, poly(3-alkylthiophene)s (P3ATs) are given priority because of their improved solubility, fusibility, and luminescence, as compared to polythiophene;^32^ hence, in our calculation we use poly(3-methylthiophene) (P3MT), which is a P3AT with the shortest alkyl side chain. A number of studies show that the energy gap of P3ATs should be similar,^17,33^ which justifies the use of P3MT.

## II Methodology

### Choice of Candidate Oligomers

In this paper we focus on poly(3-methylthiophene) (P3MT). P3MT has similar electronic properties to P3HT (see also the section reporting our test calculations), while the computational cost becomes significantly reduced because P3MT does not have the long hexyl side chains.^29^ A list of the electron-withdrawing and electron-donating substituents used here is summarized in Figure 1; the related monomers are sketched as structural formulas. The latter are arranged from left to right and top to bottom in order of increasing effect, i.e., from weak to strong electron donating and from weak to strong electron withdrawing. As it is shown in Figure 1, the methyl group, appearing on the top left of the P3MT monomer, is kept in position to preserve the properties of P3MT; then in the position occupied by the symbol R, we have substituted the original hydrogen atom with electron-withdrawing and electron-donating chemical groups.

In physical organic chemistry, it is common practice to use Hammet parameters as a quantitative measure of a functional group’s electron-withdrawing and electron-donating strength.^35^ In this work, we use four Hammett parameters representing different combinations of inductive and resonant effects,^36^ and we report them in Table 3. We then study the correlation of the four parameters as independent variables, while the HOMO–LUMO gap and HOMO and LUMO energies are the dependent variables. The results are shown in Table 2. Finally, we study the dependence of the energy, measured in electron volts, on steric hindrance.

### Overview and Strategy

The purpose here is to carry out first-principles calculations that will enable the screening of candidate donor polymers to be used as organic solar photovoltaic devices. By first-principles calculations, we mean calculations that can be accomplished before the candidate polymeric materials—or even short oligomers of these polymers—have ever been prepared in a lab. The concept is that calculations would be completed on a library of candidate materials and then, based on the screening process, only the most promising would be synthesized in a lab, characterized, and fabricated into practical devices. Several significant approximations are employed. First, we use electronic structure methods to calculate the properties of single oligomers to judge the properties of the bulk crystalline solid of the corresponding polymer. As detailed below, the HOMO and LUMO energies and the bandgap calculated for finite oligomers will be extrapolated to the long chain limit. This approach neglects the interactions between polymer chains in the bulk solid. Second, the molecular structure methods employed are relatively low level, small basis set calculations.

Even so, these oligomers containing hundreds of atoms are very large for first-principles molecular orbital calculations. Here, we use hybrid density functional methods for which the computation time scales as *N*_*e*_,^4^ where *N*_*e*_ is the number of electrons. Then, the band gap is calculated in the orbital approximation from the HOMO and LUMO orbital energies, *E*_*g*_^*hl*^ = *E*_*LUMO*_ – *E*_*HOMO*_. Finally, we calculate the inter-ring torsional potential in candidate oligomers to judge the tendency of the corresponding polymer to adopt the planar lamellar crystal structure^8,28−31^ needed to produce extended conjugation and high conductivity. In fact, we need to go beyond the structure of the pure donor polymer to consider the nanoscale structure of the donor polymer in the bulk heterojunction of a practical device.

### Choice of Molecular Structure Method

All calculations are performed using density functional theory (DFT) with the B3LYP exchange–correlation functional and 6-31G(d,p) basis set.^37,38^ Similar calculations reported in the literature mostly use 6-31G(d)^39,40^ without the p-polarized function, which is included here to improve the treatment of torsional potentials and hydrogen bonding. We also included dispersion corrections, since B3LYP does not long-range electron correlations that are responsible for van der Waals forces.^41^ The calculation level then becomes B3LYP-GD2/6-31G(d,p), and all the calculations were done using Gaussian 09.^42^

**Figure 2:**
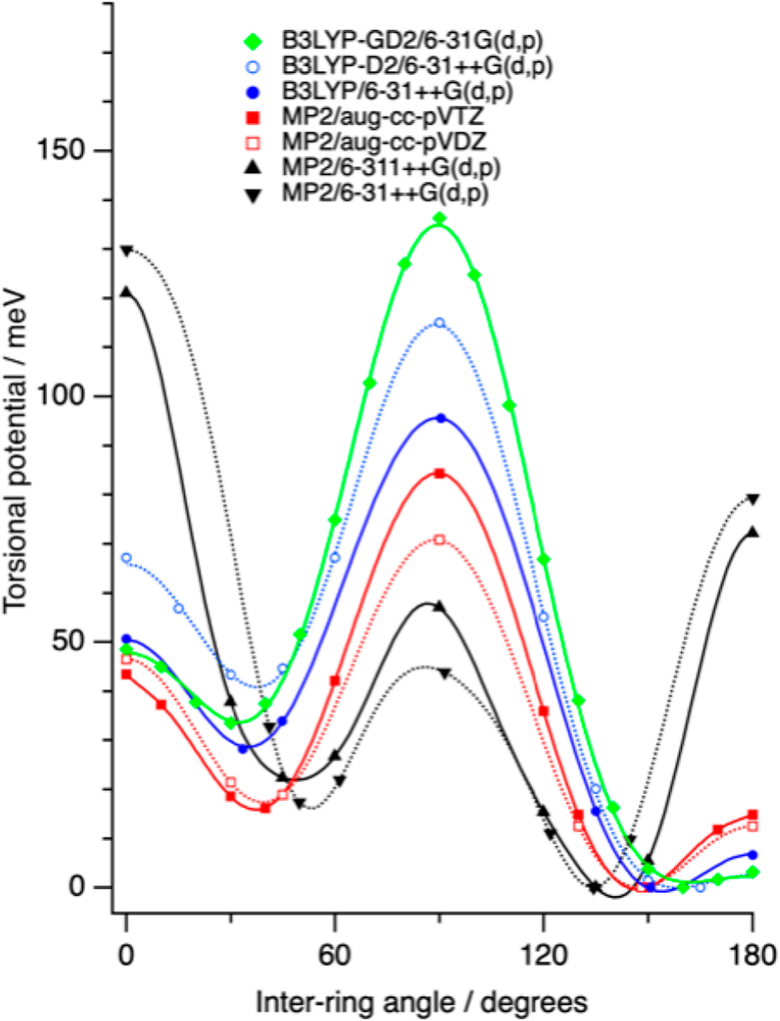
Bithiophene torsional potential versus Torsional angle for different methods. The current method is represented by the green curve with full circles. MP2/aug-cc-pVTZ from ref (43). All the other functionals are from ref (29).

Except for the inclusion here of dispersion corrections, the calculations reported in this work follow the pattern of the P3HT and P3MT oligomer calculations by Bhatta et al.^28,29^ In the present work with a series of chemically different substituents, nonbonded interactions are more varied and are expected to play a significant role in the different candidate oligomers. Those nonbonded interactions include attractive dispersive interactions as well as repulsive interactions, which one might categorize as steric hindrance. To test the acceptability of the B3LYP-GD2/6-31G(d,p) level of calculation, comparison is made with higher level calculations on 2,2′-bithiophene (Figure 2). Bithiophene is a benchmark system containing conjugated thiophene rings for which Raos et al.^43^ have completed calculations at different correlation levels (MP2, CCSD, CCSD(T), and B3LYP) and with a systematic exploration of correlation-consistent basis sets. They judged that the MP2/aug-cc-pVTZ gave the best overall description of the torsional potential (full red line with filled squares in Figure 2), and thus those calculation form the point of reference in Figure 2 for comparison of the lower level methods.^29,43^ The B3LYP-GD2/6-31G(d,p) torsional potential (green diamonds in Figure 2) shows some quantitative differences from the MP2/aug-cc-pVTZ potential, mainly a higher barrier at 90°.

Nonetheless, these two potentials share the same qualitative features: the highest energy at 90°, a lower barrier to the conformation (0°), and the rather small barrier to the trans conformation (180°).

The barrier to the trans conformation is of most interest in this work because that barrier will limit the ability of candidate oligomers to form an all-trans planar lamellar crystal structure.

Note that some more expensive higher level calculations (e.g., MP2/6-311++G(d,p),^29^ black line with downward triangles) badly overestimate the barrier to 180°.

Since we only use the torsional potentials for screening out unsuitable candidates, we expect that the torsional potential calculation does not need to be fully accurate to screen out the most unsuitable candidate oligomers. The task of these calculations is to identify and eliminate from consideration the candidate oligomers that have a high steric hindrance that would prevent them from adopting the trans conformation.

### Estimation of HOMO and LUMO Energies and the Bandgap

**Figure 3:**
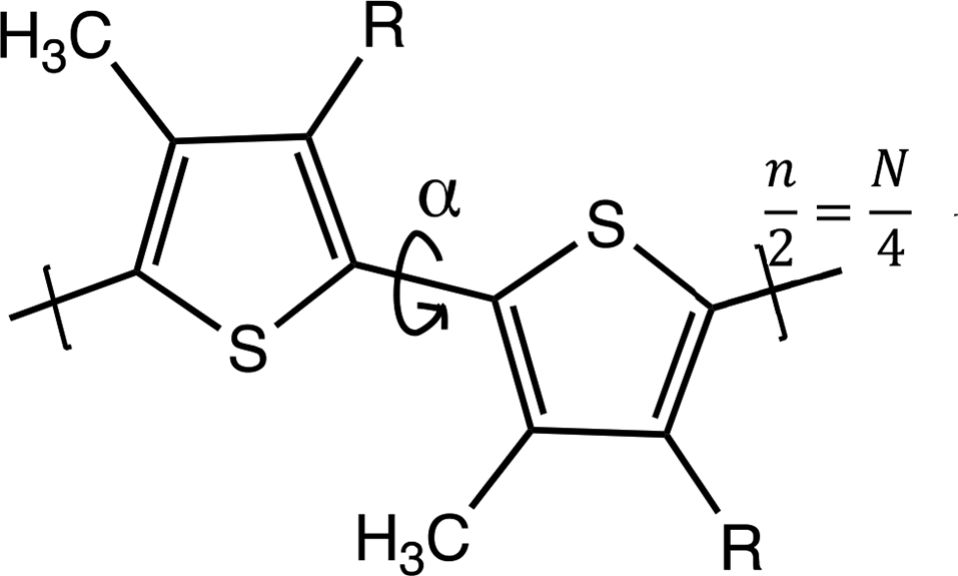
Dimer of modified P3MT. The inter-ring torsional angle α is controlled by constraining the S–C–C–S dihedral angle.

The P3HT polymers, which have been used in solar photovoltaics, form a planar lamellae structure when crystallized. In this structure, all adjacent pairs of rings are in a trans coplanar geometry. This allows extended conjugation and provides the high electrical conductivity needed in the donor material. If the thiophene-based candidate donor materials are to be effective, they too will need the extended conjugation derived from the trans coplanar geometry. Accordingly, the all-trans coplanar geometry was enforced by constraining the S–C–C–S dihedral angle between adjacent rings to be α = 180° (Figure 3).

The oligomer energies were minimized relative to all other nuclear degrees of freedom in a partial optimization calculation. All oligomer rings were found to be coplanar in the resulting structures.

The HOMO energy *E*_HOMO_ for each oligomer was taken in the orbital approximation, to be the energy of the highest occupied molecular orbital in the partially optimized structure. Likewise, the LUMO energy *E*_LUMO_ was taken to be the energy of the lowest unoccupied molecular orbital from the same calculation. The bandgap of that oligomer is approximated as *E*_*g*_^*hl*^ = *E*_*LUMO*_ – *E*_*HOMO*_.

**Figure 4:**
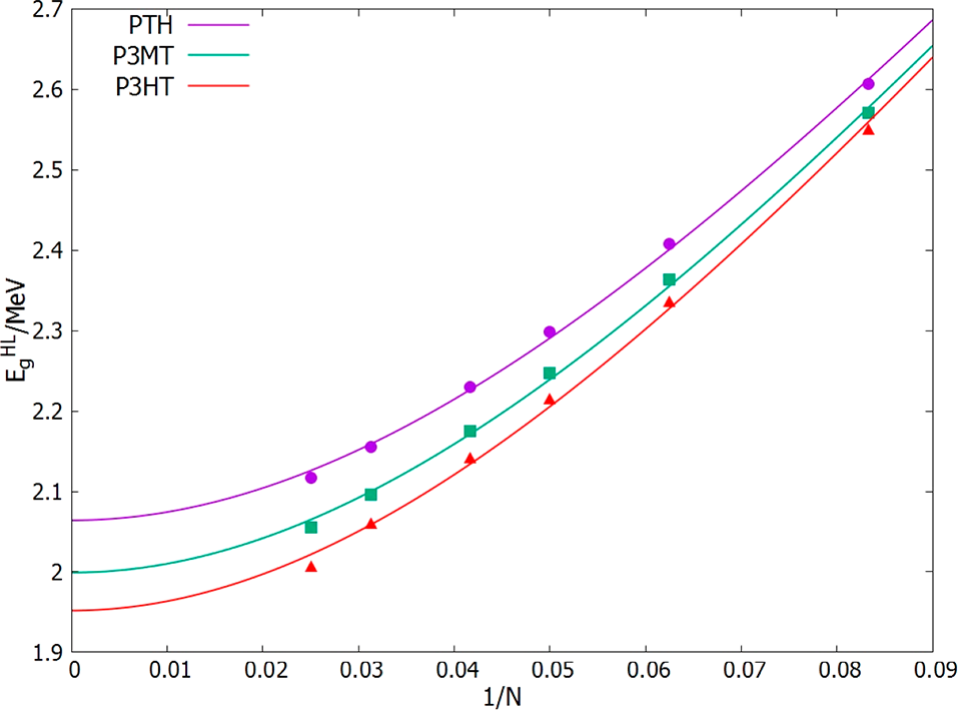
HOMO–LUMO energy gap of PT, P3MT, and P3HT calculated at B3LYP-D2/6-31G(d,p) plotted against the inverse of the number of double bonds of the backbone. Kuhn’s fit is reported as a solid line.^44^

P3MT is a polymer with approximately hundreds of repeating units; hence, it would be computationally very difficult to handle with first-principles calculations. A generally accepted approach is to obtain electronic information on short oligomers and then extrapolate computed properties to the long chain limit.^40^ This is, for example, achieved by plotting the energy band gap against , where *N* is the number of conjugated double bonds along the oligomer backbone.^8^ For thiophenes, the number of monomer units in a given oligomer is *n* = *N* /2. The extrapolation to infinite chain length is performed by using Kuhn’s method,^44^
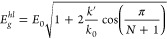
1where *E*_0_ and  are constants. The quantities, *E*_*HOMO*_, *E*_*LUMO*_, and *E*_*g*_^*hl*^ are computed for the lengths *n* = 6, 8, 10, 12, 16, and 20. The computation start with *n* = 6 repeating units and proceed with only even *n* to minimize the end-of-chain effects.^41^ Taking  as a constant, a 2-parameter fit of eq 1 is performed and the long chain limit of *E*_*g*_^*hl*^ becomes
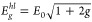
2The frontier orbital energies, *E*_*HOMO*_ and *E*_*LUMO*_, are similarly extrapolated to the long-chain limit. To benchmark to our calculation method, we performed test calculations on PT, P3MT, and P3HT. Solid crystalline PT has a known experimental HOMO–LUMO energy gap = 2.00 eV.^45^ Our calculations give a PT energy gap of 2.06 eV, which is remarkably within 3% of the value for the polymeric solid. This good agreement is likely the result of a fortuitous cancellation of errors because we used a low-level, small basis set calculation on an isolated oligomer. The same calculation was done by Bhatta and co-workers^31^ for P3HT, and it was found to have a value of 1.90 eV for *E*_*g*_^*hl*^, whereas in the present calculation we find an energy gap of 1.96 eV. Both of these calculated estimates fall within the range of published experimental bandgaps for solid P3HT films, 1.90 eV^46^ to 2.04 eV.^47^ A further discussion of the relationship between calculated and experimental P3HT bandgaps is contained in ref (31). A comparison of P3MT bandgap calculations with PT and P3HT is shown in Figure 4. As might be expected, the effect of the methyl substituent is intermediate between the hydrogen substituent in PT and the hexyl substituent in P3HT, and the bandgap obtained for P3MT is *E*_*g*_^*hl*^ = 2.00 eV.

Even if the absolute agreement with experimental bandgaps might be fortuitous, the ability to predict shifts in the value of *E*_*g*_^*hl*^ among related systems is reasonable and meaningful to our study, where we look for systematic shifts in *E*_*g*_^*hl*^ among the candidate donor materials that have the same extended-conjugation structure, but with a range of different electron-withdrawing and electron-donating substituents.

### Barrier to Coplanarity of Adjacent Rings

**Figure 5:**
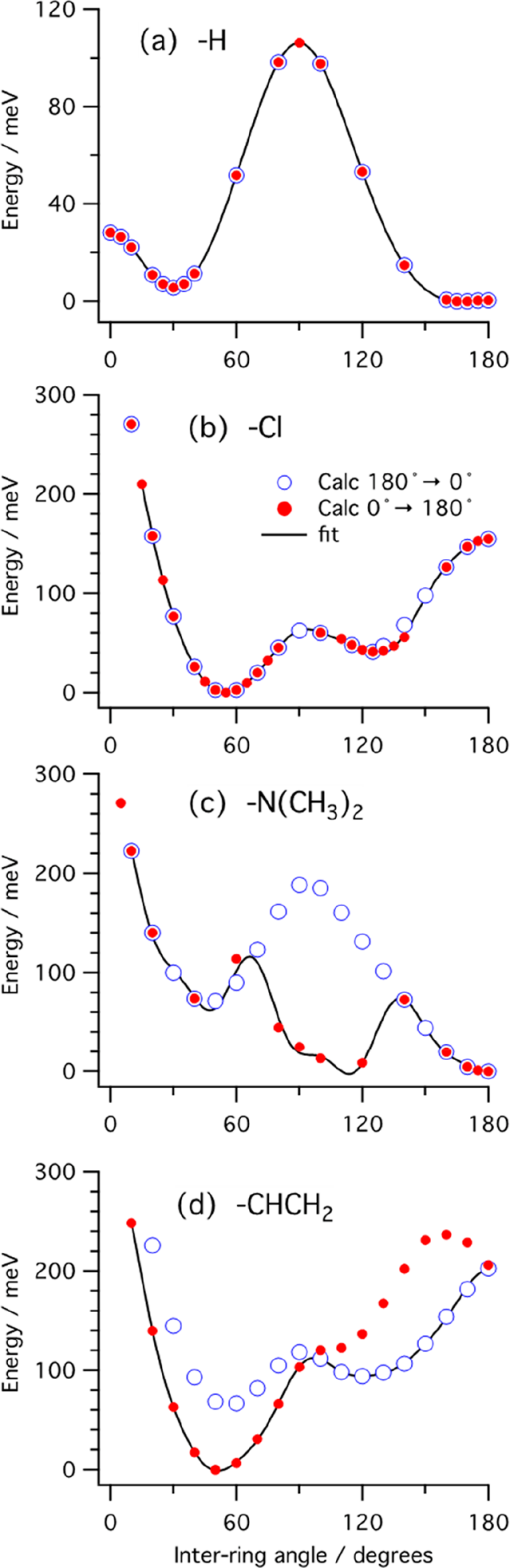
Torsional potential for central backbone angle of P3MT octamer, with all other backbone angles fixed to 180°. The fit curve serves as a guide to the eye, reflecting the lowest computed energy at each torsional angle.

In order to estimate the barrier to coplanarity between adjacent rings in a candidate donor polymer, we calculate in the inter-ring torsional potential following the procedure of Bhatta et al.^30^ The underlying assumption of this approach is that any candidate donor polymer that would be potentially useful in a practical solar device will crystallize into a planar lamellar structure in which all adjacent rings are in a trans orientation to each other. In our calculations of the bandgap, all inter-ring torsional angles α in the test oligomer chains are constrained to 180° but the middle torsion angle is varied systemically between 0° and 180°. For each selected torsional angle, a partial optimization is carried out to minimize the total energy relative to all other internal coordinates. To approximate the torsional potential for the variation of one inter-ring torsional angle in an infinite polymer chain, the test oligomers on which these calculations are carried out should be aslong as possible. In practice, it was found that the torsional potential for polythiophene converges to the long-chain limit by *n* = 8.^30^ Accordingly, the calculations of the inter-ring torsional potential for candidate donor polymers were carried out on octamers. The result of each partial optimization is then two trans coplanar tetramer fragments linked by a central inter-ring bond constrained at a specified torsional angle. The total energies of each conformation, when plotted against this central inter-ring torsional angle, yield a torsional potential energy curve (Figure 5). The challenge of this geometry optimization procedure^48^ is that it follows a steepest descent path in conformation space until it reaches a local minimum in energy. Depending on the starting geometry for the optimization, that local minimum may or may not be the conformation of the global energy minimum consistent with the applied geometric constraints. In the present situation, there is only one substituent R (on the left-hand ring of Figure 3) which may experience steric hindrance with parts of the adjacent ring, when the angle α is changed to different constrained values. For the simple functional groups in Figure 1, an experienced organic chemist would easily identify the small number of different substituent conformations that might lead to potential energy minima, and then appropriate calculations could be carried out to determine which has the lowest energy. However, for the present purpose, we wish to have a self-standing procedure that can be applied to a suite of new candidate polymers without any hinging upon professional chemical insight. Therefore, we conduct two sets of calculations, each a different starting geometry for the partial optimization.

For one set of calculations, we begin with the all-trans conformation that was optimized and used for the bandgap calculations described above. The energy of that conformation is the torsional potential energy for α = 180°. Then a “scan” is conducted whereby α is decreased in steps to 0° with a partial optimization completed at each step. This means that the initial geometry of each partial optimization is the optimized geometry that resulted from the previous step. For example, if the step increment is Δα = −10°, then the starting geometry for the optimization at α = 170° is the optimized geometry for α = 180°. The second set of calculations is conducted similarly except starting at the cis geometry α = 0° and proceeding in step increments to α = 180°. Following the example above with now Δα = +10°, the starting geometry for the optimization at α = 170° in the second set of calculations is the optimized geometry for α = 160°. Several examples of the torsional potentials resulting from these two sets of calculations are given in Figure 5. Where the two calculations give different energies at a given torsional angle, the lower energy is adopted as our best estimate of the torsional potential energy at that point. In a couple of cases (−H and −Cl), the two calculations converge to the same energy at every torsional angle investigated. For the other two cases shown in Figure 5 (−N(CH_3_)_2_ and −CHCH_2_), one of the calculations is lower in energy than the other at certain torsional angles. This reflects the fact that for one of the calculations, the functional group (Figure 1) has become trapped by a part of the adjacent ring and gotten stuck in a secondary minimum. Such calculations were completed for all 13 of the candidate polymers defined by Figure 1. In this study, the barrier to the trans planar conformation is defined as *V*_*B*_ = *V*_180°_ – *V*_*min*_, where *V*_*min*_ is the lowest energy on the potential curve (Figure 5) that can be reached from the trans conformation (α = 180°) without crossing a region that is higher in energy than the trans conformation. The purpose of this calculation is to identify donor candidates that have a substantial barrier to the all-trans conformation, which are thus unlikely to crystallize into planar lamellar structures with extended conjugation, to eliminate them as credible candidates. For this to be part of an effective screening process, the torsional potentials and the barrier heights do not need to be completely accurate, so long as many of the molecules inherently unable to form a planar crystal structure with extended conjugation can be identified and eliminated from the candidate pool. Two of the examples in Figure 5 (−Cl and −CHCH_2_) have large barriers to the trans conformation. These barriers—in the range 150–200 meV—are large compared to the average thermal energy (*k*_B_*T* = 25 meV at 300 K) so it is unlikely that packing forces would be sufficient to stabilize a planar lamellar crystal structure.

## III Results and Discussion

### HOMO–LUMO Gap and HOMO Energy

We observed that the slope of the energy gap reported in Figure 4 is relatively similar at every point, which suggests that short oligomers with small band gap might also have small gap at infinity (long chains).

We then calculated HOMO–LUMO energy gaps of monomers of the designed molecules and assumed that the above hypothesis would continue to hold. The results are shown in Table 1 for the HOMO–LUMO energy gap of modified monomers; e.g., −CHCH_2_ represents the modified P3MT as shown in Figure 3.

**Table 1:** Calculated Parameters for Different Substituents

substituent	*E*_*g*_^*hl*^ (eV)	*E*_*HOMO*_ (eV)	*E*_*LUMO*_ (eV)	*V*_*B*_ (meV)	Hammett σ_*p*_^34^	dipole moment (Debye)
–NHCH_3_	2.015	–4.13	–2.09	125	–0.84	1.56
–N(CH_3_)_2_	2.08	–3.91	–1.82	0.0	–0.83	1.51
–NH_2_	1.91	–3.74	–1.8	214	–0.66	2.13
–OH	1.99	–4.33	–2.33	135	–0.37	2.35
–OCH_3_	2.025	–4.1	–2.07	42	–0.268	1.71
–CHCH_2_	2.08	–4.22	–2.13	203	–0.04	0.63
–H	2	–4.3	–2.3	0.4	0	0.95
–F	1.97	–4.52	–2.55	0.0	0.062	0.99
–Cl	2.18	–4.82	–2.64	155	0.227	1.29
–CONH_2_	2.13	–4.8	–2.67	361	0.36	2.75
–COCl	2.19	–5.49	–3.3	228	0.61	3.56
–CN	2.14	–5.66	–3.51	38	0.66	3.81
–NO_2_	2.1	–5.73	–3.63	218	0.7	3.95

**Figure 6:**
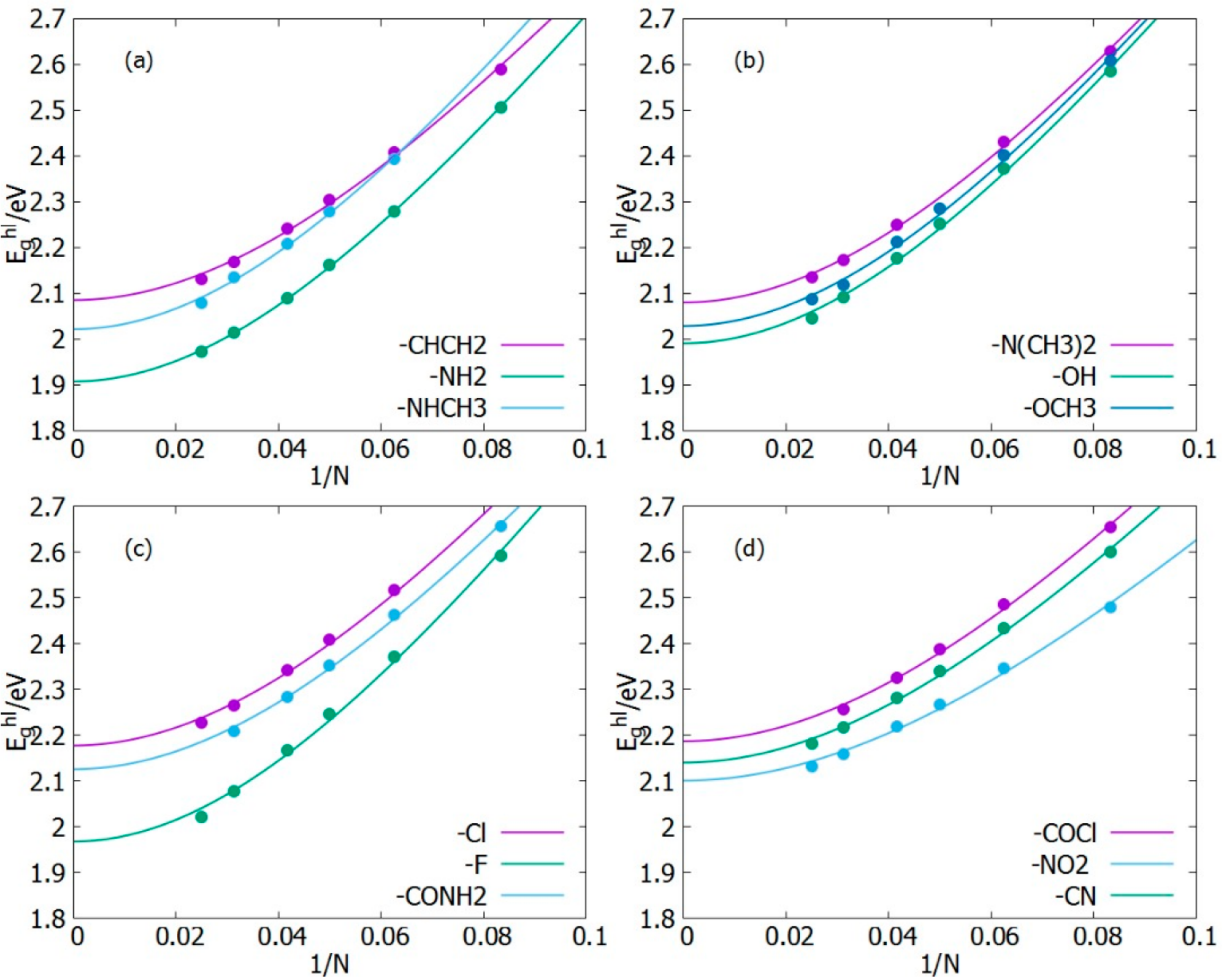
HOMO–LUMO energy gap of designed molecules plotted against the inverse number of double bonds in the backbone of oligomers chain.

Designed oligomers with electron-withdrawing groups were considered first in the top panels of Figure 6, which shows data points of the HOMO–LUMO energy gap of all the designed molecules plotted against corresponding inverse number of double bonds for oligomer length *n* = 6, 8, 10, 12, 16, 20. The graphs are fit lines, which are then extrapolated (using Kuhn’s method) to 1/*N* = 0, which corresponds to infinite length. The graphs all show a monotonic decrease of *E*_*g*_^*hl*^ with an increase in the chain length. The graphs also show as a persistent feature that, regardless of the chain length, if an oligomer has a smaller *E*_*g*_^*hl*^ than another one for the same value of 1/*N* (small *N*), that feature will hold also for oligomers (large *N*), since most of the curves have the same shape and do not cross each other.

However, there are some exceptions, as, for instance, P3MT-F which starts with a very high energy gap of 2.59 eV and P3MT-NO_2_ which starts with an energy gap of 2.48 eV; we observe that at long chains P3MT-NO_2_ ends up with a higher energy gap than P3MT-F. A similar behavior is shown when comparing P3MT-Cl and P3MT-COCl. The results are reported in Table 1.

**Figure 7:**
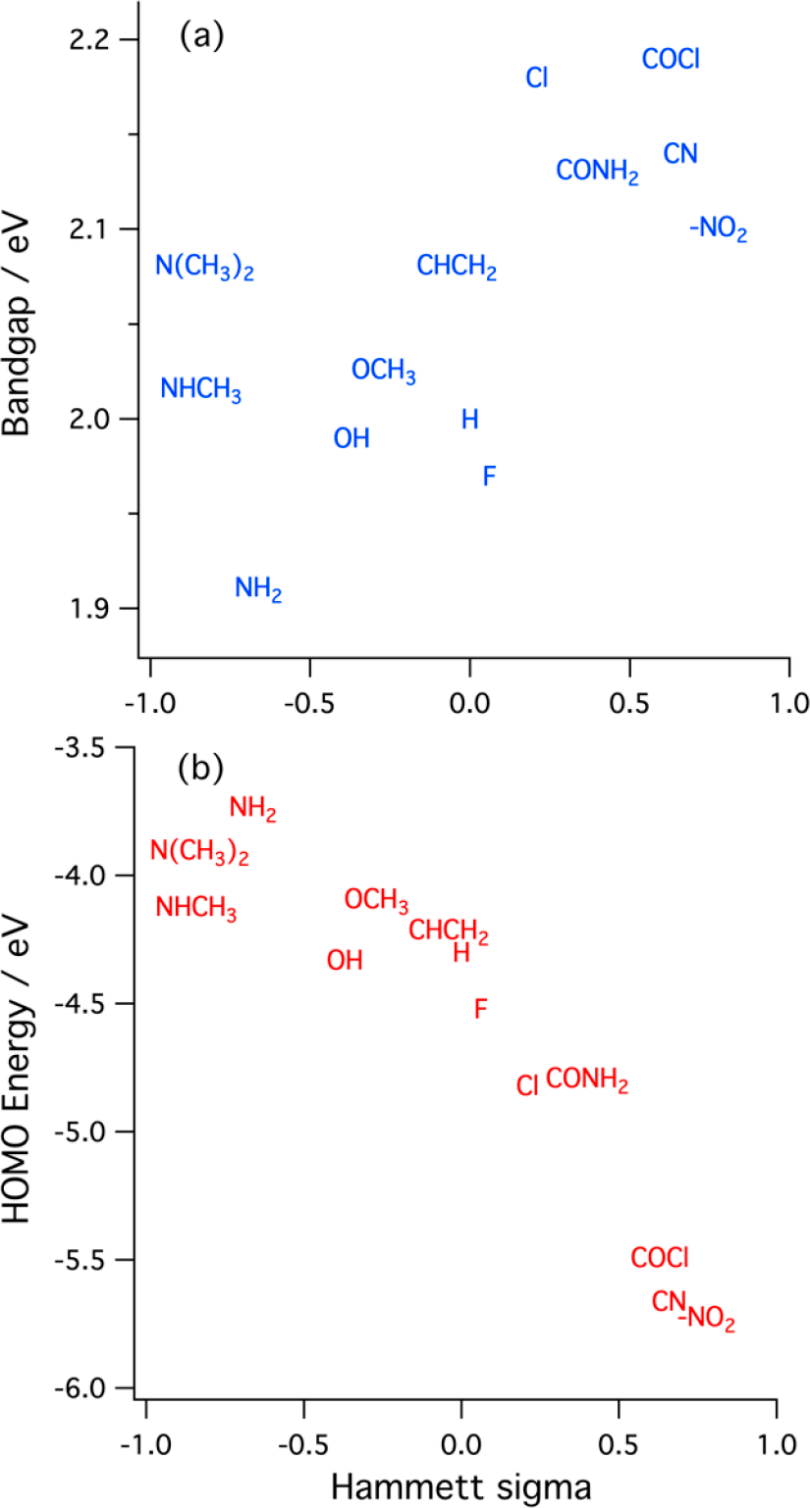
Calculated bandgap (a) and HOMO energy (b) plotted versus the Hammett sigma parameter for each substituent.

The results reported in Table 1 show that adding electron-donating groups generally favors lower energy gaps than adding electron-withdrawing groups, with a highest and lowest energy gaps of 2.10 and a 1.91 eV, respectively, while electron-withdrawing groups exhibit highest and lowest energy gaps of 2.19 and 1.97 eV, respectively. However, the correlation of the HOMO–LUMO gap with the Hammett parameter σ is rather weak (Figure 7, Table 2). The parameter σ quantifies the influence of the substituent on the electronic distribution of the reactive center of the molecule due to both inductive and resonance effects.

**Table 2:** Correlation Coefficients of σ_*p*_, *R*, *F*, σ_*m*_, and Steric Hindrance (Φ) Used as Independent Variables and the HOMO–LUMO Gap and HOMO and LUMO Energies Used as Dependent Variables

	bandgap	HOMO	LUMO
σ_*p*_	0.66	–0.87	–0.91
*R*	0.70	–0.80	–0.86
*F*	0.46	–0.85	–0.83
σ_*m*_	0.60	–0.90	–0.91
Φ	0.32	–0.08	–0.20

Based on the current calculation, the absolute lowest energy gap is shown by the −NH_2_ substitution. Our data indicate that *E*_*g*_^*hl*^ does not depend just on the electron-donating or -withdrawing strength, especially when you compare P3MT-F and P3MT-Cl, which possess a similar electron affinity. In fact, P3MT-F gives a gap of 1.97 eV while P3MT-Cl gives 2.18 eV, showing a nontrivial difference of 0.21 eV.

The knowledge of the energy levels of frontier orbitals is also important to understand the performance of organic photovoltaic devices.^49^ A low-lying highest occupied molecular orbital is desirable to enhance open circuit voltage (VOC).^50^ VOC is also affected by the morphology of the active layer because a large donor/acceptor interfacial area is associated with a high charge carrier recombination, and VOC losses.^51^ The open circuit voltage is defined as the maximum voltage a solar cell can provide to an external circuit,^19^ which directly contribute to the OSC PCE through the following equation:
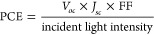
3where *J*_*sc*_ is the short circuit current density and FF is the fill factor. Table 1 shows the HOMO energies of our molecules. It emerges quite clearly that a higher value of the Hammet parameter *σ_p_*, which is by increasing the electron-withdrawing strength of the substituent, the HOMO energy shifts down, and the lowest HOMO energy of −5.73 eV was found when −NO_2_ is used as a substituent.

### Steric Hindrance to Coplanar Rings

As mentioned before in finding the steric hindrance for the systems being studied here, we used the octamers of each system. All backbone torsional angles were constrained to 180°, while the middle torsional angle (α), which separates the oligomer into two halves, was varied from 0° to 180° (forward calculations) and from 180° to 0° (reverse calculations). We first observed that some molecules had different energy levels at some points for the two calculations. There are many factors that can contribute to this feature, but the most likely factor could be molecules getting stuck in some local minimum. In cases where the results of the forward and reverse calculations were different, the lowest energy was taken to be the best estimate of the minimum energy. Figure 5 shows examples of the calculations, the first two are for −H and −Cl which had the same minimum energy for forward and reverse calculations while the last two are for −N(CH_3_)_2_ and CHCH_2_ which clearly produced different results. The torsional potential exhibits more than one minimum across the systems. The steric hindrance is therefore calculated as the difference between the energy at 180° and the energy at the global minimum. The results are shown in column 5 of Table 1. F, N(CH_3_)_2_, and OCH_3_ show 0 meV steric hindrance, which makes them better candidates for solar cell materials. Using PTB7 as a reference, which has a PCE greater than 10%, with a torsional barrier as defined above of <≈170 meV, we set that value as an upper limit for acceptable barrier. The results given in Table 1 show that most of our molecules fall within the range, except for NH_2_, COCl, CHCH_2_, and CONH_2_. Since the value of the molecular dipole moment can be related to the dissociation efficiency of the exciton (provided the value assumed in the excited state is known), in the last column of Table 1 we report the ground state values of it.

Finally, we studied the correlation of the four Hammet parameters and the steric hindrance with the HOMO–LUMO, HOMO and LUMO. Table 2 shows a very small correlation of −0.08 for HOMO, followed by −0.2 for LUMO and 0.32 for the band gap with the steric hindrance. The results in Table 2 also imply that the band gap and the HOMO and LUMO energies strongly depend on σ_*p*_ and *R*, but the low correlation with respect to gap suggests that there are other factors that are contributing which are not taken into account by the Hammet parameters. The complete list of Hammet parameters used in this work is reported in Table 3.

**Table 3:** Substituents Used to Modify the Basic P3MT, with Their Hammett Parameters (σ_*p*_, *R*, *F*, and σ_*m*_) and Steric Hindrance (Φ)

	σ_*p*_	*R*	*F*	σ_*m*_	Φ (meV)
–NHCH_3_	–0.84	0.03	–0.73	–0.21	125
–N(CH_3_)_2_	–0.83	0.15	–0.98	–0.16	0.0
–NH_2_	–0.66	0.08	–0.74	–0.17	214
–OH	–0.37	0.33	–0.70	0.12	135
–OCH_3_	–0.268	0.29	–0.56	0.12	41.5
–CHCH_2_	–0.04	0.13	–0.17	0.06	203
–F	0.062	0.45	–0.39	0.34	0.0
–Cl	0.227	0.42	–0.19	0.37	155
–CONH_2_	0.36	0.26	0.1	0.28	361
–COCl	0.61	0.46	0.15	0.51	228
–CN	0.66	0.51	0.15	0.56	38
–NO_2_	0.7	0.65	0.13	0.71	218

HOMO and LUMO energies are reasonably well correlated (−0.8 to −0.91) with the Hammett parameters. The bandgap is also loosely correlated with the Hammett parameters. By contrast, there is little, or no correlation at all of the bandgap and HOMO or LUMO energies with the steric hindrance.

### Opportunity for Rational Design

**Figure 8:**
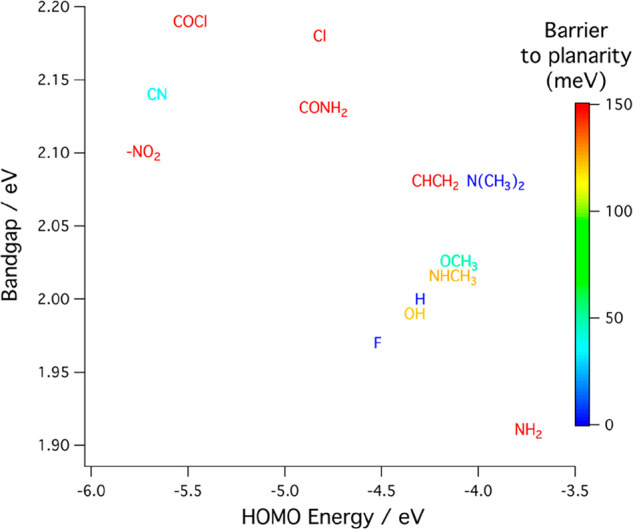
Band gap and HOMO energy with different with the barrier to planarity shown by color.

Figure 8 summarizes the results obtained for the three computed single-oligomer properties as obtained for each of the 13 candidate oligomers. The rational design concept presented here is to use these computed properties to screen candidate donor materials. First, the bandgap determines the wavelengths within the solar spectrum that are absorbed by the proposed photovoltaic device, that is, which part of the solar spectrum might be converted into electricity. In a device with two or more stacked heterojunctions, one wants to choose the absorption maxima appropriately for each of them. Second, the HOMO energy level, in conjunction with knowledge of the bandgap, determines the alignment of the energy levels between the donor and acceptor materials. One wants a slightly downhill path for the electron transfer to provide a driving force, but not so far downhill that a substantial fraction of the available photon energy is dissipated as heat. Finally, the steric hindrance to a coplanar ring structure should be small or zero.

If the barrier to coplanarity between adjacent rings is large, e.g., greater than *k*_*B*_*T*, then it is unlikely that packing forces between oligomers will be able to overcome this barrier to form a planar lamellar crystal structure, which is needed to obtain the maximum extended conjugation and good electrical conductivity.

The single crystal packing forces are weakest at the edges of the crystal, that is, at the interface between the donor and acceptor in the bulk heterojunction. At these critical interfaces, even a moderate barrier to coplanarity between adjacent rings can result in torsional disorder that degrades the material properties. The modest correlation between the bandgap and the HOMO energy that is evident in Figure 8, means that, within limits, one can select a donor material that has the desired combination of these two properties. The barrier to coplanarity of adjacent rings is indicated by the color scale on the right scale. The oligomer in blue, or perhaps also those in blue-green, have a minimal barrier height and hence are most likely to form planar lamellar crystal structures. The others are unlikely to crystallize easily or to have good electrical conductivity.

## IV Conclusions

We have demonstrated a possible approach to the rational design of donor materials for organic solar cells, which is based on DFT calculations on single oligomers with electron-donating and electron-withdrawing substituents. These calculations are extrapolated in the long chain limit and can be successfully used for estimating the potential of the polymer material in practical solar devices. These are “first-principles” calculations in that they can be conducted with no prior knowledge of each candidate material and on candidate materials that have never been synthesized in the laboratory.

We have focused on three single-oligomer properties of P3MT: (i) the bandgap *E*_*g*_ that determines the light frequency that would be absorbed, (ii) the HOMO energy, which for given *E*_*g*_ controls the alignment of the HOMO and LUMO energies of the donor material relative to the acceptor, and (iii) steric hindrance to coplanarity, which controls the ability of the material to stack in a robust planar-lamellar fashion.

When a hydrogen atom of P3MT is substituted with −NH_2_, −OH, and −F, we obtained a HOMO–LUMO energy gap that is less than the one of P3MT (2.00 eV). Our calculations show that the bare electron-withdrawing or electron-donating strength cannot even be used to predict the ordering of the energy gaps in the candidate oligomers (Figure 7(a)). Substitution with −NH_2_ reduces the HOMO–LUMO energy gap of P3MT by ∼0.1 eV, while the lowest lying HOMO level is obtained for NO_2_, suggesting that in a situation where HOMO is the relevant parameter to be tuned, −NO_2_ is the best substituent. If we consider as a potential acceptor the standard fullerene derivative PCBM, we find that all exhibit an appropriate LUMO level since it is 0.3 eV higher than the LUMO of the acceptor; i.e., there will be enough energy for allowing the exciton to dissociate at the interface between the two materials.^20^ COCl, CONH_2_, and NO_2_ give an undesirably high steric hindrance. Thus, our study indicates that it may be worth implementing prescreening methods, such as empirical force field structure optimizations, to eliminate the cases of severe steric hindrance. In general, the followed procedure allowed us to identify the substituents with a band gap suitable for successful exciton dissociation, at the interface between our target donor polymer and the standard acceptor PCBM: −F, −NH_2_, and −OH give the best compromise between the band gap, HOMO, LUMO and steric hindrance.

While the low-level DFT calculations that we performed may not be accurate, we believe they are still able to predict qualitatively the shifts of the properties we considered when the substituents are inserted systematically in a generic oligomer. On the other hand, the parallel computation required substantial CPU time on a cluster of computers. The prospect of both linear scaling methods, massively parallel calculations on GPUs instead of CPUs, as well as the optimization^14^ or development of new DFT functionals^52^ means that large screening and combinatorial design of candidate polymers will become possible. Even if we limited our study to a donor (conjugated^53^) polymer and to a restricted number of properties to be investigated, a similar screening may be applied to acceptor materials, including small molecules, with a different or wider range of microscopic observables.

## Notes

Data are available on request from the authors.
